# Difficulties and motivations for physical exercise in women older than 65 years. A qualitative study

**DOI:** 10.1590/1518-8345.2392.2989

**Published:** 2018-07-16

**Authors:** Yolanda López-Benavente, José Arnau-Sánchez, Tania Ros-Sánchez, Mª Beatriz Lidón-Cerezuela, Araceli Serrano-Noguera, Mª Dolores Medina-Abellán

**Affiliations:** 1 RN, Servicio Murciano de Salud, Centro de Salud Vistabella, Murcia, Murcia, Spain.; 2 PhD, Researcher, Servicio Murciano de Salud, Centro de Salud Vistabella, Murcia, Murcia, Spain.; 3 Researcher, Servicio Murciano de Salud, Centro de Salud Moratalla, Murcia, Murcia, Spain. Scholarship holder at Ministerio de Educación, Cultura y Deporte, Spain.; 4 PhD, Professor, Facultad de Enfermería, Universidad de Murcia, Murcia, Murcia, Spain.; 5 RN, Servicio de Urgencias de Atención Primaria de Abarán, Abarán, Murcia, Spain.; 6 PhD, MD, Servicio Murciano de Salud, Centro de Salud Espinardo, Murcia, Murcia, Spain.

**Keywords:** Gender, Health, Aged, Exercise, Motivation, Research

## Abstract

**Objective:**

to identify difficulties and motivations for the practice of physical exercise in women older than 65 years.

**Method:**

qualitative study based on the phenomenological theory, with focus groups and in-depth interviews. The nursing staff selected 15 women by intentional sampling using the following criteria: age, time dedicated to physical exercise, independence, and absence of cognitive impairment and contraindication for this activity. Two focus groups were formed (one of them did physical exercise for less than 150 minutes per week and the other at least 150 minutes per week) in addition to conducting five in-depth interviews. Qualitative analysis of the data was performed through transcription, coding, categorization, and verification of results.

**Results:**

the difficulties to start and develop physical exercise were circumscribed to the perception of poor health and lack of free time; both circumstances result from care obligation, being represented as a gender imposition. However, the motivations are related to perception of strength, need for socialization, and perception of autonomy and freedom.

**Conclusions:**

the ideological representation of gender determines the women’s decision to exercise. Knowing the meaning and significance that women give to health and their role in the socio-family environment allows nurses to develop relationships and interventions to encourage the practice of physical exercise.

## Introduction

Regular physical exercise (PE) and gender are determinants of mortality and active or successful aging^1^ ^-^ ^3^. In addition, PE is an important predictor of quality of life^4^ ^-^ ^5^. Although its benefits are known, most people with advanced age does not participate in PE^6^ ^-^ ^11^.

Sedentary lifestyle is the fourth most important mortality risk factor worldwide, causing both the appearance of chronic degenerative diseases and installation of incapacitating processes^12^ ^-^ ^13^. Some international studies confirm that women of advanced age are more sedentary than men of the same age group^14^ ^-^ ^17^. The National Health Survey in Spain (2011-2012) indicates that 58.4% of the population with more than 65 years old referred to be sedentary, with a higher percentage in women (63.6%)^13^, and 52% of those over 16 years of age lived in Murcia^18^. 

The gender role and family responsibilities are the main causes of non performing PE^19^ ^-^ ^20^. According to the Institute for Women in Spain, these findings lead us to suppose that 17.3% of older women do not exercise^21^. 

Most of the female population does not have the time necessary to dedicate to occupations not related to the procreation role. Therefore, the activities developed in the social life do not have as much importance as those derived from gender impositions. The wife, mother, and caregiver roles obtain more social value and time than activities related to PE or recreation^22^. 

If sport, which follows a model of androcentric values, is added to this lack of time and valuation of PE, the barriers for women to open a space for such activity are increased. The PE is so oriented to men, that women have to understand it within the male model and “re-socialize” to be integrated, accepting these customs that do not take into account half the population^23^. 

For this reason, it is important to explore these socially-established conceptions and design interventions that promote health and physical activity, considering the gender perspective^14^ ^,^ ^17^ ^,^ ^24^. 

**Figure 1:**
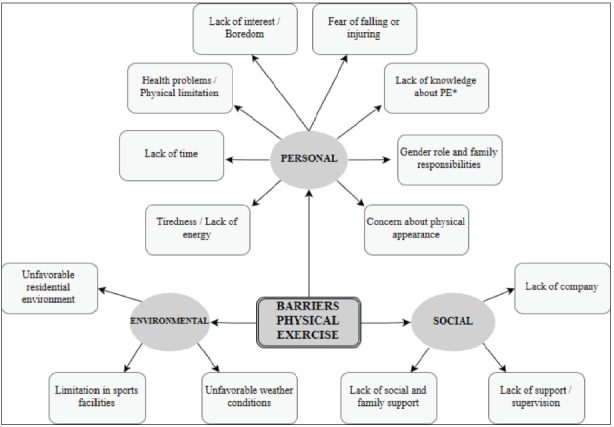
Map of Barriers to do Physical Exercise. Prepared by the author from a literature review

**Figure 2:**
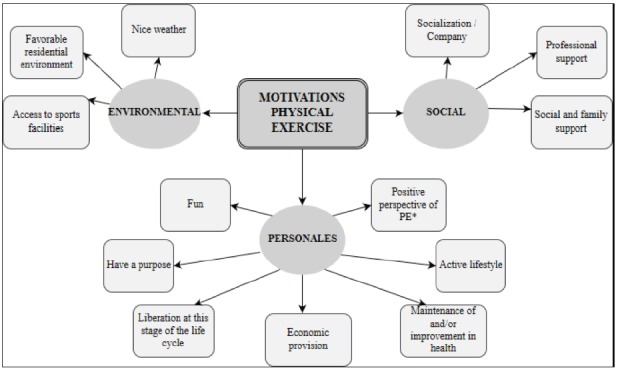
Map of Motivations to do Physical Exercise. Prepared by the author from a literature review

We conducted a literature review of the existing scientific evidence about studies related to barriers and motivations to do PE. Eight references were then included: six reviews (systematic or integrative)^8^ ^,^ ^19^ ^-^ ^20^ ^,^ ^25^ ^-^ ^27^ and two original articles^7^ ^,^ ^9^. In Figure 1 (Map of Barriers to do PE) and Figure 2 (Map of Motivations to do PE), we can see the result of this review and better understand how personal, social, or environmental factors can act motivators or barriers.

*PE: Physical Exercise

*PE: Physical Exercise

Reducing sedentary lifestyle and promoting access to PE to obtain an active aging in older women, from a consultation of the Primary Care (PC) Nursing, is expected to provide opportunities to choose healthy lifestyles, within the expectations of older adults^28^. 

For this reason, our objective was to identify and understand the difficulties and motivations for the practice of PE in women over 65, in a Basic Health Area (BHA), taking into account the following aspects of this group: personal experiences, idiosyncrasies of health, family, and sociocultural context^29^. 

## Method

This is a qualitative study based on the phenomenological theory. In November 2015, two focus groups (FG) were formed as a tool for data extraction. They were organized as a function of the weekly time dedicated to PE as recommended by WHO^6^. A group of 8 women who did PE for ≥ 150 minutes (min) per week (FGA) and another group of 7 women who did PE for < 150 min per week (FGB). A thematic guide was used as a stimulus for the debate^30^, which contained the following aspects: a) what they understood by doing PE; b) benefits of PE; c) motivations to do it; and d) difficulties in doing it. 

**Figure 3:**
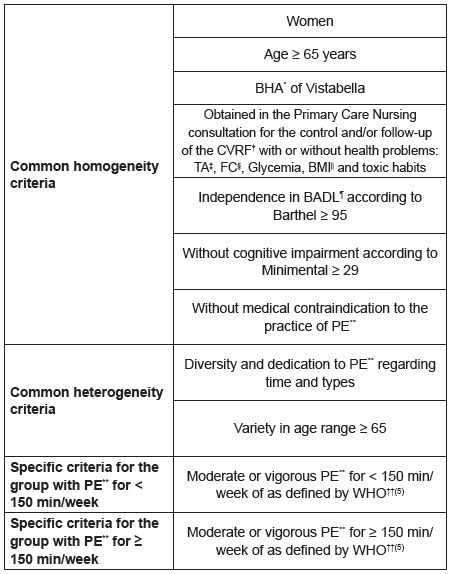
Inclusion criteria. Murcia. Spain. 2015

The inclusion criteria were circumscribed to homogeneity and heterogeneity, as shown in Figure 3. 

*BHA: Basic Health Area †CVRF: Cardiovascular Risk Factors ‡BP: Blood Pressure §HR: Heart Rate ||BMI: Body Mass Index ¶BADL: Basic Activities of Daily Life **PE: Physical Exercise ††WHO: World Health Organization The number within parentheses refers to the bibliographic citation

The total sample comprised 15 women with ages in the range 65-82 old, selected by key informants during AP Nursing consultation, in a Basic Health Area (BHA) of Murcia, which received and signed the Patient Information Sheet and the Informed Consent Form. 

The FG meetings were held at the Neighborhood Association of Vistabella, and the participants did not know the moderator who prevented influencing the discourse^31^. 

At the end of speech analysis of both FG, the appearance of categories that were not initially conceived was observed. These emerging categories related the barriers, which the participants felt to perform PE in the FGB, with gender impositions. Because in-depth knowing this information was considered important^31^, it was decided to carry out (in a meeting room of the health center; April 2016) five in-depth interviews (IDI) with five women from that group who revealed additional information about such relationship. 

Once all statements were recorded and the data were transcript, we proceeded to analyze its content. The information obtained was segmented into basic units of meaning (coding process). Subsequently, these units were grouped into broader categories (categorization process). The transcripts were coded by four researchers in an independent way. At all times, anonymity and confidentiality of participants’ identity were assured^31^. The MAXQDA10 software was used to analyze the data. 

**Figure 4:**
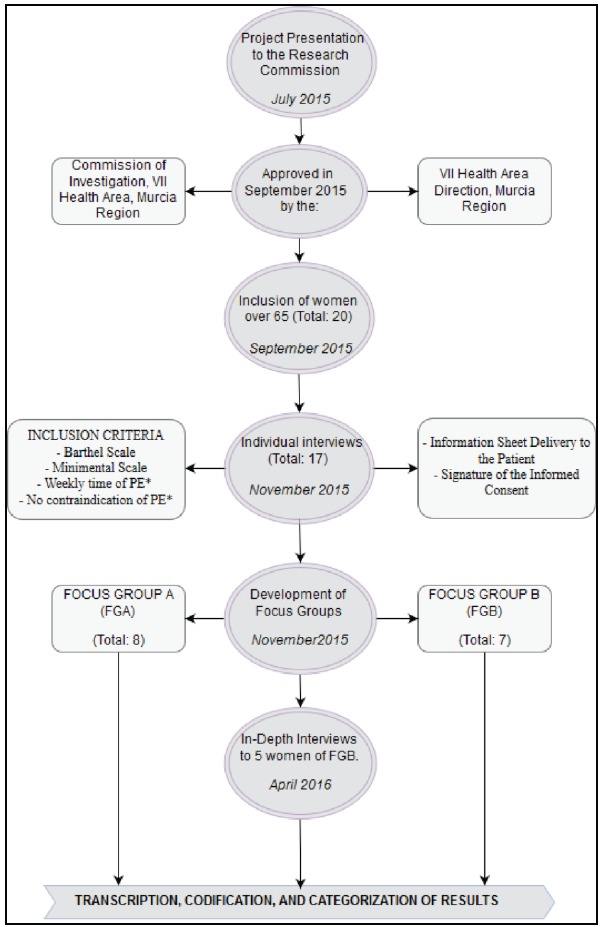
Methodological Procedure used in this study

The methodological procedure followed in this study can be observed in figure 4. 

*PE: Physical Exercise

The study was approved by the Research Commission, and approval was given by the Direction of the Reina Sofía Hospital, Area VII of Health, Murcia-East, Murcian Health Service. This study also had a Collaboration Scholarship from the Ministry of Education, Culture, and Sports, course 2015/16. 

## Results

From the FG and PE speeches, three large categories were obtained for women over 65: ideological representations of PE, interference with PE, and motivation and predisposition to PE. 

Regarding the ideological representations of PE among women over 65, the study participants understood PE in different ways: on the one hand, some women expressed that doing housework is supposed to be a physical activity, without being understood as PE; on the other hand, other women represented the concept in a broader way describing it as that physical activity done in a gym or outdoors, like hiking or walking; these understandings are expressed in the following statements.

*I also believed that, at home, women could do housework, that everything would have an effect, when making the bed you would have to bend the knee, not your body … and it would also help us. In fact, I believe [this] has been very useful for women to live longer because we move at home …(FGB05). I understand physical exercise as an activity that is done outside housework, because... everything that is done outside home, anywhere you do an exercise, it seems to me physical exercise and good, that has an effect on the person. Or it can be hiking, or it can be walking, it can be tai chi as it is said... It can be any gymnastics, anything that is... yoga, for example, it is also a physical exercise, that isn’t as strong as gymnastics, but it is also good. Anything like that...(FGB08).*

So that, the women in this study do not have a unanimous position regarding activities that can be understood as PE and those that are not PE. 

Among different difficulties in PE in women over 65, sense of insecurity and body limitations were found as perceptions of bad health state. 

Some women manifested a perception of their health as bad, which generated lack of self-confidence to do PE and doubts about flexibility and response of their bodies during its practice. Their own negative stereotypes associated with old age generate insecurity due to an excessive concern about their abilities.

*Right now, I am afraid to go to yoga because I know I have to throw myself to the ground …. But it is because of the kilos I have, (…), that I am a very active person, but the ground pulls me back. (…). So, difficulty for PE is also according to the flexibility you have, I have it very little (FGA08). The older I am, the more ridiculous I look and… I have to go, I that didn’t look ridiculous, I know … But I have had a complex, physical and… of being good for nothing, and well… of everything. A thousand complexes. A thousand complexes that I shouldn’t have suffered (IDI03). If I climb stairs with three steps, and the other day I almost fell, I already begin to think that I can’t go up the stairs anymore because I am going to fall… All those things that are limiting you, and this is changing me. I don’t see myself anymore as I was. I don’t see an exit. (…) It isn’t that I don’t value myself as a person, is that I already see myself very… that I am shrinking my… my physical abilities and at the same time… and if the head affects me, then also psychic capacities, I know (IDI06).*

It is worth highlighting the convincing effect of the negative influence that changes, which are linked to the aging process, exert, since they do not favor doing physical exercise, rather, they interfere with such activity decreasing women’s confidence in their own abilities. 

The care obligation and gender imposition are other interferences we found; these women were educated to care. The participants have internalized the caring role as an obligation, sacrificing a large part of their free time and dedicating it to care tasks; these are not only linked to domestic tasks but also to the care of a relative who is disabled or suffering from some disease. Regarding this, the caring role is very much embedded in the collective imaginary of women, considering that they are prepared and educated to take care of the family since they are born; this is a socially and culturally imposed obligation. Such circumstance obliges them to dedicate a large part of their time to care, to the detriment of PE. All this has a negative effect both to start and do PE. The participants mention it in this way.

*Walking, now I walk a lot less, … among grandchildren, sons, daughters-in-law who come four days to eat... in the afternoon, I am busted. (…) You never take time to go… We don’t love ourselves too much, my husband says. He says: you love others, and you go leaving. (…) I also see it, if you don’t need it, then tell me no. I see it, that I love others more than me (FGB03). I used to play handball, basketball, and even I did swimming…, I walked a lot and went to the gym, but I came to take care of my mother three years ago and what I practice now is armchair and sandwich (…) (FGB06). (…) That I notice it, that I do, that… that I prefer… to stay for the best wanting to do something and give it to other (IDIB03). I don’t see life anymore… I see it as shorter, shorter, you know? A very short time to be as my mother is. And then on the other hand I think: no, why can’t you start doing exercise that this is very important to me, you know? (…) With my mother, I drown, I am drowning (IDIB06).*

Thus, the participants stated an important assumption of the caretaker role, which determines their priorities to benefit people who are meaningful to them. 

Motivation and predisposition of women over 65 years to do exercise is the third category of our results. Among them, we find the maintenance of the optimal state of health, the perception of strength, and coping with aging. 

When women reach this stage of life, they perceive some inability to remain active due to the aging process. From this preconceived idea, they develop strategies to feel encouraged to practice PE; they start from the knowledge they have about the health benefits that performing this activity is supposed to bring them. This is the main engine on which they rely to maintain their strength and improve their health status.

*I think that physical exercise is health, because it is good to lower those who have cholesterol, good for tension and obesity, and good mentally and physically. So that... physical exercise is good for everything, to extend our life too (FGB08). At first, when I was detected to have a little high blood pressure, I was advised to go for walk. I had never done it, but it was a plan to walk. But then I started to activate myself a bit and first, because I felt really tired, because walking isn’t the same as leaving with a slightly stronger rhythm. I became accustomed and now I do it much more easily, and I have been two or three times in some gymnastics group. Now I am also in an Elders’ Association twice a week and the other days I go for an hour to walk (FGA02).*

Older people are convinced that doing PE brings benefits to all health dimensions. 

The need to relate with other people is another factor that influences the motivation to initiate and develop PE. On the one hand, it contributes to create a network of social relationships and to further strengthen those already established; and on the other hand, a company while doing PE gives them security and distraction. Accompaniment is so relevant that it can become a barrier to start and develop PE when it is not available.

*I think that physical exercise is health, because it is good to lower those who have cholesterol, good for tension and obesity, and good mentally and physically. So that... physical exercise is good for everything, to extend our life too (FGB08). I have a neighbor who can’t walk, the poor, because her bones are bad, and of course, in the past we went both, we go there, talk, have a coffee, and go around, but now, as she can’t do it, How do I go alone?.. (FGB05). What I want is to live life, but with a group of people that make me laugh, have a good time, have fun, that’s it. In the sense of that, of being happy, of going, for example, to walk or hike, I would also like to… I know, there are things that you like. And there, in those places, friendships are made and… And well, as I see it well, the head is also cleared (IDI06).*

It is clear that besides the benefits in the mental and physical dimension of health the participants identify advantages in the social dimension, which acts as motivation, both to be initiated in PE and to maintain its practice. 

Most participants in the discussion group concerning women who do PE highlight the feeling of freedom and autonomy, in this stage of the life cycle, as an impulse at the time of doing PE. The fact of feeling free to perform any activity, without limitations imposed by other people or pre-established schedules, showed to be of great importance to motivate them to do PE.

*It makes me easier that, as I am alone, I go where I want and how I want. (…) If they must propose it. That in the mornings I get up and say: Oh, now…. If this is to be proposed (FGA09). This is the freedom that I said… And we are freer. (…) I, for example, I am also a widow for 8 years… And when the cold starts, as I own myself, if instead of leaving at 8, I want to leave at 11:30 to take sun, then I do it (FGA03). Since I retired… I yes, I now yes, yes, yes. Of this. I am, really, I don’t depend on anyone. I solve everything, you know? (IDI07).*

It is understood that for these people, being able to go at their own pace is an element that adds to others, increasing the appeal of developing a physical activity. 

## Discussion

For the women who participated in the study, PE is represented in a polysemic way. While for the group that did not exercise regularly any activity developed in the domestic sphere was understood as PE, for the group that developed PE regularly, it was understood as a planned and organized activity, that took place outside of the family home, not related to domestic tasks. This way of conceptualizing PE agrees with the concept that the international and Spanish organizations have; they emphasize the idea that PE should be planned, structured, and done on regular basis, aiming to maintain or improve the physical conditions^32^ ^-^ ^33^. 

Despite this definition, there is a widespread conviction among women that “any physical activity they perform would fit within the PE concept” and, as such, would contribute in a beneficial way to their health. 

The perception of a bad state of health, which women with advanced age have, is supposed to be a great limitation to start and maintain PE. The women who participated in the study perceived limitations in their own bodies, which generated a great distrust in the response that it would have after doing PE. With reference to the above, some international investigations highlight the power of negative stereotypes associated with old age and their connection with PE. These same studies emphasize that, in the context of physical activity, women represent old age as a lack of adequate response of the body to face the challenge of PE; this would be due to a decrease in their physical abilities^7^ ^,^ ^19^ ^-^ ^20^ ^,^ ^34^. However, in this BHA, the ideological representations of senescence and limitations that it imposes to the body, did not present as a great obstacle to start a new activity. Differently, other studies point out the slowness of the recovery process after doing PE and the concern about suffering injuries in this age group as the main barriers to its development^35^. 

Gender is another analysis category that emerges in the results of this study. The statements show that women were educated and socialized during their maturity to take care of previous and future generations by cultural and social imposition of gender^36^, which leads them to identify themselves as a “being for others” and “omnipresent”, defining them as health preservation agents of the other members of the family. This circumstance makes them adopt a role that is difficult to change, not only by the cultural force, but also due to the conflicts they suffer when they try to break the behavior rules imposed by culture. Therefore, they are driven to respond satisfactorily to the expectations related to the care and attention to others that the family has of them^37^ ^-^ ^38^. The women expressed it this way while arguing that they continued frequently helping with feeding their children’s family or taking care of grandchildren, thus reducing their interest in starting and developing PE. 

Based on the above, the Women’s Institute spoke about it arguing that the fact of having to take care of other people drives both men and women to practice less or not to practice sport, and the negative effect was greater on the female gender^21^. Although women can hardly be free from the sociocultural pressure that the duty and imperative of parenting and care assigns them^39^, the participants stated that the obligation to do housework gives them satisfaction, since they perceive that it is about roles that were acquired and transmitted from generation to generation, thus providing them with a deep well-being and, therefore, they did not express any need to do some type of PE. 

Despite the perception of disability, limitations of their own bodies, and the barriers imposed by the ideological representations of gender at the beginning and development of PE, women strive to maintain this activity because of the positive perception they have about the benefits produced by PE. In this regard, some Spanish and international studies confirm our findings highlighting health as the main reason to recommend physical activities; thus, these studies are a motivating element for women to practice PE and initiate themselves in sports, activities that acquire more relevance as they age^7^ ^,^ ^20^ ^-^ ^21^ ^,^ ^35^ ^,^ ^40^. It is reasonable to assume that, although elderly women recognize their bad health, their perception of feeling able to do PE and the feeling of well-being (when they overcome the limitations of their own bodies) contribute to their initiation and continuity in PE. 

Inversely, other international investigations claim that despite the benefits brought by PE, it has not power enough to start and sustain this activity^41^. 

According to the factors that motivate the development of PE in elderly women, their need to socialize is a remarkable influence, both to start and maintain this activity. In this perspective, some studies reveal that women generally feel uncomfortable when they walk alone, feeling better when doing it with a company, as this activity allows to create and reaffirm links^7^ ^-^ ^8^ ^,^ ^42^. The importance of a companion is significant because it allows women not only to enjoy the benefit of the company itself, but also to establish and reaffirm social ties that become support and solidarity; they give meaning to their life projects in this stage of the life cycle^39^. 

The feeling of freedom and the perception of autonomy, which this stage of life is supposed to become relevant to the practice of PE because it entails a way for them to control their lives, making their own decisions, becoming a privileged moment for personal renewal; this drives to the pursuit of pleasure, satisfaction and, what is most important, allows them self-determination^36^ ^,^ ^43^. This makes them feel comfortable with their reality and with themselves. Similarly, the study of the Women’s Institute also establishes a relationship between freedom, autonomy and physical exercise, by stating that independence of their children, health, widowhood, and availability of time for retirement are found among the reasons for women older than 65 initiate sports practice^21^. This relationship allows them take the incomes of their life, being freer and happier at this moment of their life cycle^39^. On the other hand, in the group of women who do less exercise, the responsibility of family care, a consequence of being a connection between the previous and subsequent generations^44^, decreases freedom and autonomy to do exercise due to the absence of free time. 

Regarding the limitations of this study, we understand that people who agreed to participate in this study surely were those who collaborated most. Considering that we intend to know the phenomenon in depth, probably the group of women who were less motivated to participate could contribute with very relevant information. Similarly, it is possible that use of a recorder has caused some interference in the speeches, limiting or blocking spontaneity in some participants. On the other hand, the fact that the investigation was conducted in a specific BHA decreases the possibility of generalization to other areas and communities, our objective was to deeply know the difficulties and motivations of elderly women for PE. 

After analyzing the difficulties and motivations of PE in women older than 65, the results found by the investigation allow us to know the meaning these women give to taking care of their health and doing exercise, as well as their interaction with the family and social environment, from a gender perspective. This knowledge will be useful for nurses to develop relationships and interventions according to the needs and particularities of these women, which will help them overcome gender grievances. Therefore, the study will encourage a deeper reflection among health professionals about the categories of analysis related to aging, gender, health, and PE. 

## Conclusion

In our time, women over 65 have been educated within a culture in which providing care to others has priority over caring for themselves; in addition, the gender imposition associated to housework and serving as a wildcard for other generations hinders the articulation of self-care and the practice of PE. Although the above-mentioned aspects and the perception of a deteriorated health is an important barrier to start and develop PE, the factors related to the need for socialization and the perception of obtaining autonomy and freedom, which women experience during exercise development, strongly impose the initiation and maintenance of PE. 
